# Bone marrow stimulation in arthroscopic rotator cuff repair is a cost-effective and straightforward technique to reduce retear rates: A systematic review and meta-analysis

**DOI:** 10.3389/fsurg.2023.1047483

**Published:** 2023-02-21

**Authors:** Lei Zhang, Yanlin Zhu, Tianhao Xu, Weili Fu

**Affiliations:** Department of Orthopedics, Orthopedic Research Institute, West China Hospital, Sichuan University, Chengdu, China

**Keywords:** rotator cuff retear, bone marrow stimulation, microfracture, arthroscopic rotator cuff repair, glenohumeral osteoarthritis, meta-analysis

## Abstract

**Background:**

Bone marrow stimulation (BMS) has been considered a well-established method for treating knee and ankle osteochondral lesions. Some studies have also shown that BMS can promote healing of the repaired tendon and enhance biomechanical properties during rotator cuff repair. Our purpose was to compare the clinical outcomes of arthroscopic repair rotator cuff (ARCR) with and without BMS.

**Methods:**

A systematic review with meta-analysis was performed according to the Preferred Reporting Items for Systematic Reviews and Meta-Analyses (PRISMA). PubMed, Embase, Web of Science, Google scholar, ScienceDirect, and the Cochrane Library were searched from inception to March 20, 2022. Data on retear rates, shoulder functional outcomes, visual analog score and range of motion were pooled and analyzed. Dichotomous variables were presented as odds ratios (OR), and continuous variables were presented as mean differences (MD). Meta-analyses were conducted with Review Manager 5.3.

**Results:**

Eight studies involving 674 patients were included, with mean follow-up period ranging from 12 to 36.8 months. Compared to ARCR alone, the intraoperative combination of the BMS resulted in lower retear rates (*P* < 0.0001), but showed similar results in Constant score (*P* = 0.10), University of California at Los Angeles (UCLA) score (*P* = 0.57), American Shoulder and Elbow Surgeons (ASES) score (*P* = 0.23), Disabilities of the Arm, Shoulder and Hand (DASH) score (*P* = 0.31), VAS (visual analog score) score (*P* = 0.34), and range of motion (ROM) (forward flexion, *P* = 0.42; external rotation, *P* = 0.21). After sensitivity analyses and subgroup analyses, no significant changes in statistical results were observed.

**Conclusion:**

Compared to ARCR alone, the combination of intraoperative BMS can significantly reduce the retear rates, but showed similar short-term results in functional outcomes, ROM and pain. Better clinical outcomes are anticipated in the BMS group by improving structural integrity during long-term follow-up. Currently, BMS may be a viable option in ARCR based on its straightforward and cost-effective advantages.

**Systematic Review Registration:**

https://www.crd.york.ac.uk/prospero/, identifier: CRD42022323379.

## 1. Introduction

Rotator cuff tears are one of the most common causes of shoulder pain and impaired shoulder function (1). When conservative treatment fails, the patients are recommended for ARCR to restore the anatomy of the native rotator cuff tendon insertion. Although repair techniques have evolved from single-row repair to double-row repair to transosseous-equivalent/suture bridge repair, there are still considerable retear rates. Especially for large to massive tears, the retear rates range from 30% to 64% (2, 3). The primary factor for tendon retears is the disorganized scar tissue that formed during the healing process, which failed to restore biological structure and biomechanical strength (4).

Numerous initiatives have been launched to encourage tendon-bone mending in addition to ongoing advancements in surgical techniques. Among them, biological treatments for rotator cuff repair are attracting increasing attention (5). These biological strategies have promising avenues, but challenges remain at present. Some studies have reported that stem cells can significantly decrease retear rates (6, 7), but data on long-term impacts based on human studies are rare. Adverse events associated with stem cells cannot be ignored before clinical application, such as cell leakage, the growth of tumors and administration site reactions (8). PRP serves as a most common biologic agent for the treatment of musculoskeletal disorders. However, inconsistent efficacy claims and the unknown composition of PRP formulations have restricted further clinical use (9–11).

It has been extensively reported that the BMS technique produces satisfactory clinical results in osteochondral lesions of the knee and ankle (12–14). Proposed by Snyder in 2009 (15), BMS for rotator cuff repair is drawing increasing interest due to its safety and high cost-effectiveness. Bone marrow droplets containing mesenchymal stem cells, growth factors and other elements from the drilled hole are recruited onto the repaired tendon to promote tendon-bone healing. Nevertheless, conflicting results exist concerning the efficacy of the BMS in promoting healing (16–19).

Two reviews on this topic have been published (20, 21). However, the credibility of the conclusions is compromised by applying inappropriate inclusion criteria or recruiting overlapping patient populations. Besides, several high-quality and relevant articles have been published in recent years (19, 22, 23). This study aimed to assess whether the use of BMS in the ARCR could result in additional clinical benefits. We hypothesized that applying the BMS in the primary ARCR would lead to lower retear rates, better functional outcomes and ROM.

## 2. Method

This study was reported according to the PRISMA guidelines (24). The protocol was registered at PROSPERO before starting this review (CRD42022304686).

### 2.1. Search strategy

We systematically searched electronic databases, including PubMed, Embase, Web of Science, Google scholar, ScienceDirect, and the Cochrane Library, on March 20, 2022, to identify potentially relevant studies. The literature search was performed using a search strategy with the combinations of the following items: [rotator cuff OR rotator cuff repair OR rotator cuff tear OR rotator injury OR rotator rupture] and [microfracture OR bone marrow stimulation OR marrow]. The gray literature and unpublished studies databases were also examined, as well as potentially eligible studies manually identified from the reference lists of included studies. There was no restriction on the publication date. Two reviewers independently performed literature searches, and any discrepancies were settled through discussion by the reviewers. On October 13, 2022, we repeated the search to update the search results, but no new qualifying publications were discovered.

### 2.2. Inclusion and exclusion criteria

Inclusion criteria for studies were as follows: (1) All comparative studies [randomized controlled trials (RCTs) or observational studies] of human patients undergoing primary ARCR; (2) The control group was treated by ARCR alone. The BMS group was treated by arthroscopic repair with BMS, including multiple channeling, microfractures, Crimson Duvet procedure, etc.; (3) Studies with a minimum 1-year follow-up; (4) At least one of the following outcomes was reported (retear rates, the Constant score, the UCLA score, the ASES score, the DASH score, the VAS score, ROM). Exclusion criteria were as follows: (1) Combined BMS and any augmentation for ARCR; (2) Applying BMS prior to arthroscopic surgery; (3) Nonclinical studies (e.g., cadaveric or animal model); (4) Studies with the smallest cohort or shortest follow-up (different studies focusing on the same group of patients); (5) Case reports, case series, comments, ongoing trials; (6) Studies published in languages other than English.

### 2.3. Quality assessment of individual trials

For RCTs, 2 reviewers (L.Z. and Y.Z.) independently assessed the methodological quality of the included RCTs using the Cochrane Collaboration's risk of bias tool (25). Each RCT was evaluated based on the following items: random sequence generation, allocation concealment, blinding of participants and personnel, blinding of outcome assessment, incomplete outcome data, selective reporting and other biases. The risk of bias for each item was rated as high, low, or unclear.

For non-RCTs, the same 2 reviewers independently assessed the risk of study bias and methodological quality using the methodological index for non-randomized studies (MINORS) (26). A MINORS item scored 0 if not reported, 1 if reported but not adequate and 2 if reported and adequate. Twelve items with a maximum possible score of 24 points. Comparative studies with a MINORS score of 17 or higher were considered high quality, otherwise low quality (27). Any disagreements in the quality assessment were resolved by discussion with a third reviewer (W.F.).

### 2.4. Data extraction

Two reviewers independently extracted data from eligible studies according to predefined criteria, including publication information (first author, year of publication, study design, level of evidence), patient information (sample size, age, sex), surgical procedure (method of fixation, BMS protocol), rehabilitation program and surgical outcomes (retear rates, functional outcomes, VAS score, ROM). Functional outcomes included the Constant score, the UCLA score, the ASES score, and the DASH score. If necessary, we will contact the corresponding authors of the included studies to obtain the original data.

### 2.5. Statistical analysis

This study was conducted according to the Cochrane Reviewer's Handbook, and statistical analyses were performed using Review Manager (RevMan for Macintosh version 5.3; The Cochrane Collaboration). For continuous outcomes, a generic inverse-variance method was used to calculated mean differences (MD) and 95% confidence intervals (CI). For dichotomous outcomes, a Mantel-Haenszel method was used to calculated odds ratios (OR) and 95% CI. Heterogeneity between studies was quantified by *I*^2^. *I*^2^ < 25%, 25%–50%, and >75% indicated low, medium and high heterogeneity, respectively. When *I*^2^ < 50%, the fixed-effects model was applied; otherwise, the random-effects model was used. *P* < .05 was considered to be statistically significant. Sensitivity analyses were performed by sequentially removing included studies to assess the impact of individual studies on the pooled results. Subgroup analyses according to RCT design or non-RCT design were conducted for available outcomes.

## 3. Results

### 3.1. Search results

**Figure 1:**
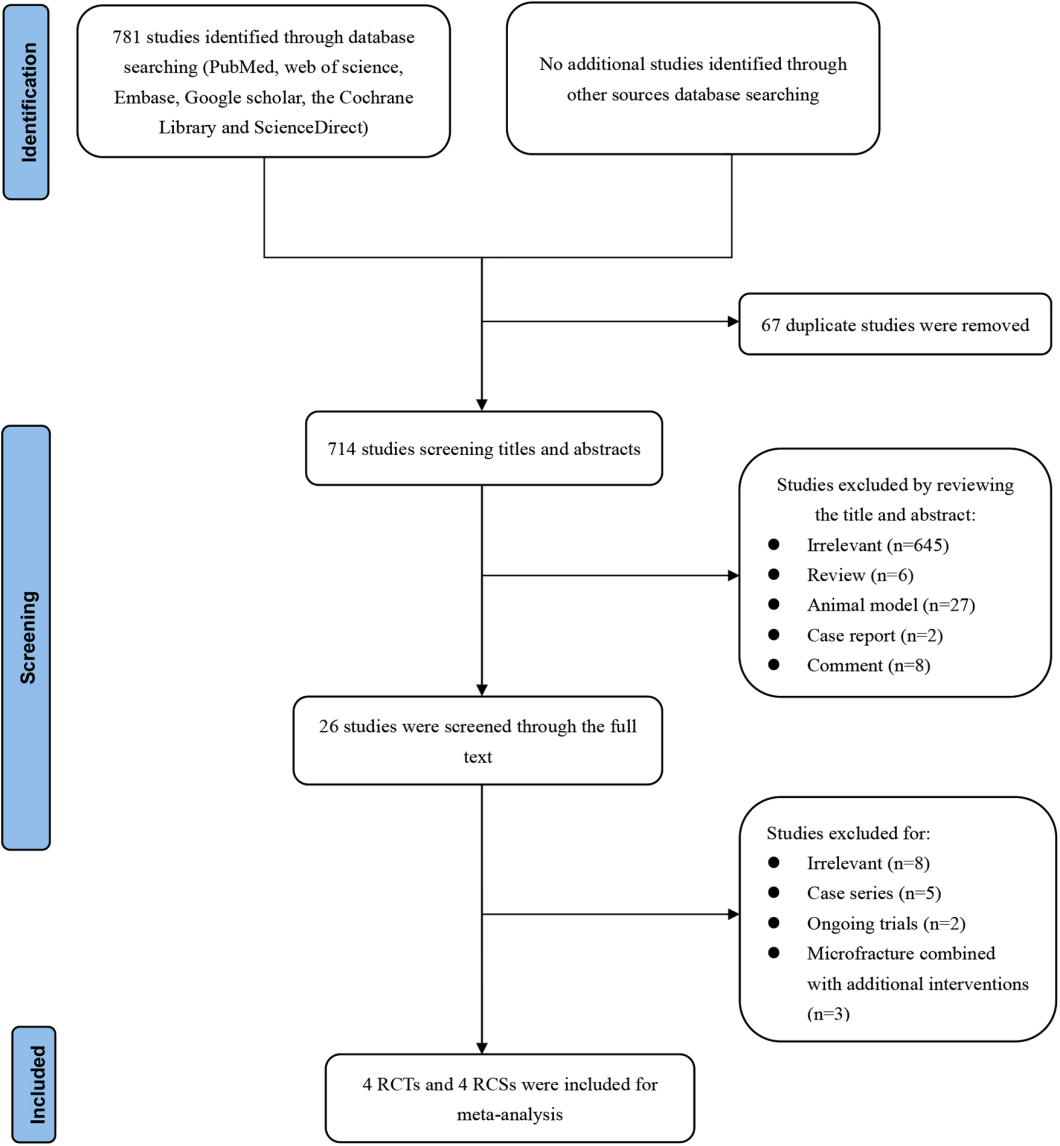
Preferred reporting items for systematic reviews and meta-analyses diagram on article selection for systematic review.

A total of 781 records were retrieved through the literature search. After removing 67 duplicate studies, we further excluded 714 based on title and abstract screening, resulting in 26 studies for full-text review. Two studies (18, 28) by Jo et al. focused on the same group of patients, and the study with shorter follow-up (18) was excluded. The study by Yoon et al. (29) was excluded because it combined BMS and patch augmentation. The study by Lapner et al. (30) was excluded because the surgeon performed the BMS technique 5 to 7 days prior to surgery, rather than during arthroscopic surgery. Ultimately, 8 articles (19, 22, 23, 28, 31–34) were included in the meta-analysis, including 4 RCTs (19, 28, 31, 32) and 4 retrospective cohort studies (RCSs) (22, 23, 33, 34). The PRISMA diagram of the article search and selection process is shown in Figure 1.

### 3.2. Basic characteristics of included studies

A total of 674 patients (354 male, 320 female) were included in this meta-analysis. Of those, 336 patients underwent ARCR combined with BMS and 338 patients received ARCR only. The mean age of patients ranged from 57.8 to 64.3 years, and the mean follow-up period ranged from 12 to 36.8 months. Six studies (22, 28, 31–34) enrolled patients with full-thickness rotator cuff tears. One study (19) included patients with large to massive rotator cuff tears. One study (23) included patients with supraspinatus tears smaller than 3 cm. As for the technique of ARCR, 4 studies (19, 31, 33, 34) used a single-row technique, 2 studies (23, 28) used double row or transosseous equivalent repair, and 1 study (32) used the surface-holding method. One study (22) selected surgical techniques based on tear size; for tears less than 1 cm, the single row technique was used, and the double row technique was preferred for tears greater than 1 cm. As for other procedures combined intraoperatively, 5 studies (22, 23, 28, 31, 33) performed biceps tenotomy or tenodesis according to the age and preoperative findings of biceps tendon integrity, except Osti et al. (34). They performed a long head of the biceps tenotomy in all instances. In addition, 4 studies (22, 23, 28, 31, 32) reported that acromioplasty was performed when necessary. We found that 1 study (31) compared three techniques, and to reduce heterogeneity, data were extracted only from the BMS group and its control group. The basic characteristics of these studies are shown in Table 1. The BMS technique and rehabilitation program are shown in Table 2.

**Table 1:** Basic characteristics of and patient demographics included studies in the analysis.

First author, year	Study design	Sample size (male), n		Patient age, y Mean ± SD		Level of evidence	Type of injury	Repair technique	Outcome measurement	Mean follow up period
		BMS	Control	BMS	Control					
Jo et al. 2013	RCS	57 (25)	67 (34)	58.89 ± 8.67	60.10 ± 7.94	III	full-thickness RC tear	Double row	Retear rate, Constant, UCLA, ROM, VAS, DASH, SST, SPADI, ASES, structural integrity	36.8 months
Milano et al. 2013	RCT	35 (22)	38 (19)	60.6 ± 10.1	63.1 ± 9.2	I	full-thickness RC tear	Single row	Retear rate, Constant, DASH, structural integrity	28.1 months
Osti et al. 2013	RCT	28 (16)	29 (13)	61.2 (38–73)	59.8 (34–71)	I	full-thickness RC tear	Single row	Retear rate, Constant, UCLA, ROM	29 months
Taniguchi et al. 2015	RCS	44 (22)	67 (42)	64.7 ± 1.4	64.3 ± 1.1	III	full-thickness RC tear	surface-holding	Retear rate, structural integrity, complication	13.7 months
Kim et al. 2020	RCS	56 (26)	42 (23)	64.6 ± 6.0	64.2 ± 5.5	III	large to massive RC tear	Single row	VAS, Subjective Shoulder Value, ASES, UCLA, ROM	24 months
Pulatkan et al. 2020	RCS	44 (11)	40 (15)	58.1 ± 9.7	59.2 ± 10.1	III	full-thickness RC tear	Single row	Retear rate, Constant, VAS	30 months
RuizIbán et al. 2021	RCT	36 (14)	33 (18)	60.1 ± 7.88	57.8 ± 10.7	I	supraspinatus tear	Double row or transosseous equivalent repair	Retear rate, Sugaya's grade, Brief Pain Inventory, Constant, EQ-5D-3L, complication	12 months
Toro et al. 2022	RCT	48 (29)	47 (25)	58.9 ± 7.7	57.8 ± 9.2	II	full-thickness RC tear	Single row or double row	Retear rate, ASES, Constant, ROM	12 months

RCT, randomized control trial; RCS, retrospective cohort study; RC, rotator cuff; UCLA, University of California at Los Angeles; ROM, range of motion; VAS, Visual analog scale; DASH, Disabilities of the Arm, Shoulder and Hand; SST, Simple Shoulder Test; SPADI, Shoulder Pain and Disability Index; ASES, American Shoulder and Elbow Surgeons; EQ-5D-3L, EuroQol Five Dimensions Questionnaire.

**Table 2:** Parameters of bone marrow stimulation technique and rehabilitation program.

First author, year	Instrument	Diameter (mm)	Depth (mm)	Interval (mm)	Site	Rehabilitation program			
						Immobilization	Passive motion	Active exercise	Strengthening exercise
Jo et al. 2013	bone punch	2.1	10	4–5	from the articular cartilage margin to the lateral ridge of the greater tuberosity	for 4–6 weeks using an abduction brace	the day after surgery for patients with small- to large-size tear; 6 weeks after surgery for massive tear	from 4 to 6 weeks after surgery	3 months after surgery
Milano et al. 2013	arthroscopic awl	1.5	5	4	the attachment area of the tendons	for 4 weeks using a sling	from 4 to 8 weeks after surgery for range-of motion exercise program (passive, active, active assisted)		9 to 16 weeks after surgery
Osti et al. 2013	arthroscopic awl	none	2–4	3–4	from the juxta-articular space to the tip of the greater tuberosity	for 4 weeks using a sling	from 2 to 4 weeks after surgery	from 6 weeks after surgery	from 12 weeks after surgery
Taniguchi et al. 2015	metal bar	3	none	3–5	along the medially advanced footprint	for 6–8 weeks using an abduction pillow	from 2 weeks after surgery	from 8 to 10 weeks after surgery	from 10 to 12 weeks after surgery
Kim et al. 2020	custom-made awl	2	10	5	the lateral half of the footprint	for 6 weeks using an abduction brace	from 6 weeks after surgery	from 8 weeks after surgery	from 3 months after surgery
Pulatkan et al. 2020	custom-made awl	1.3	5	4–5	the lateral side of the repair zone in the greater tuberosity	immobilization in 30 ° abduction for 4 weeks	started at 4–6 weeks	started in 2 months	Not reported
RuizIbán et al. 2021	NanoFx	1	9	3–5	from the articular edge to the border of the footprint	Not reported			
Toro et al. 2022	mechanical awl	none	3–5	3	the surface of the exposed footprint	Not reported			

### 3.3. Assessment of risk of bias

**Figure 2:**
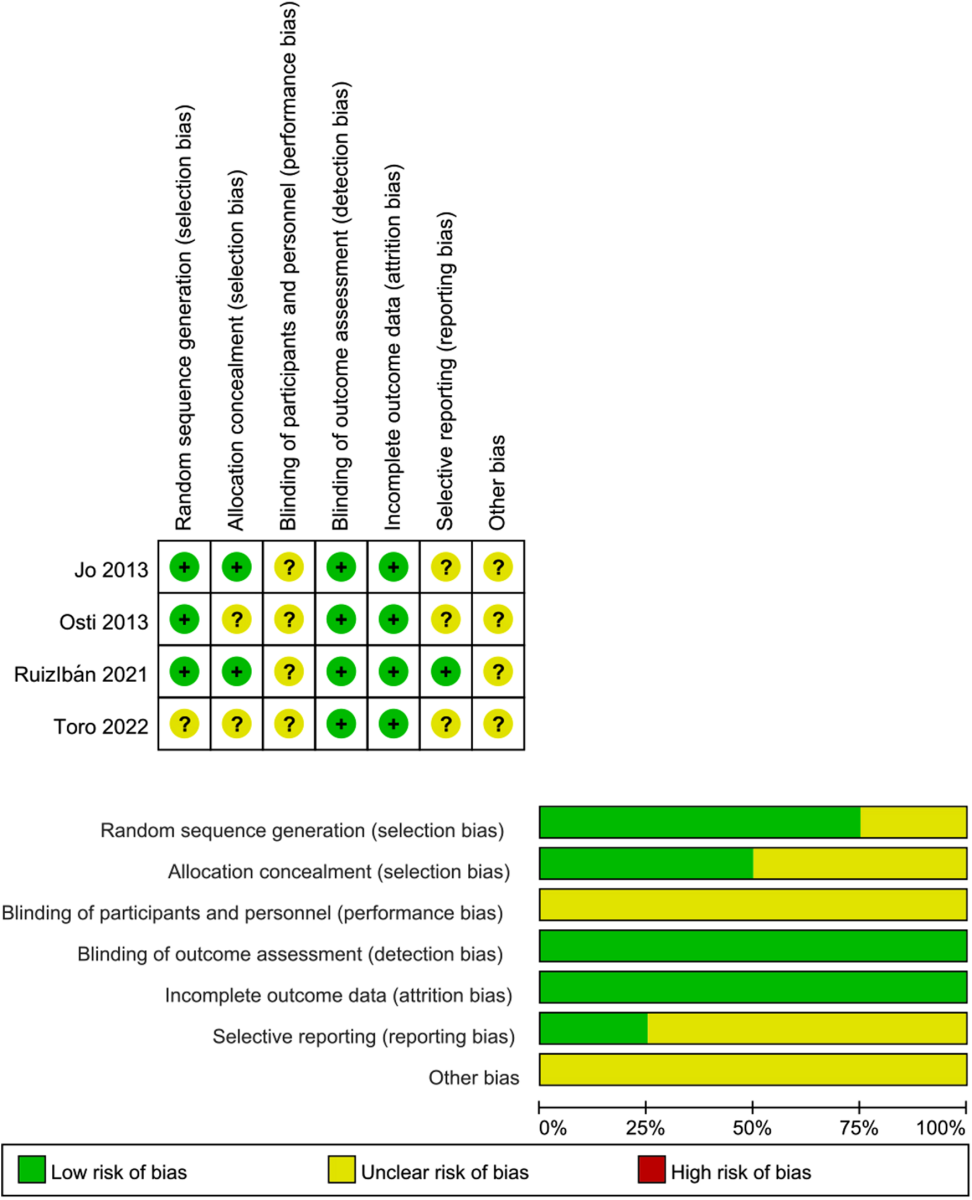
Risk of bias of included studies.

Two authors independently assessed the quality of the included studies based on the study design. The results of the risk of bias assessment on included RCTs were summarized in Figure 2. Only 1 study (22) did not clearly report the procedure of randomization and was rated as unclear risk of bias. Two studies (22, 34) did not adequately report allocation concealment and were rated as unclear risk of bias. All RCTs reported the blinding of outcome assessments and were rated low risk of bias. The results of the risk of bias assessment included non-RCTs were summarized in Table 3. The MINORS score ranged from 17 to 19, with a total score indicating good quality.

**Table 3:** Risk of bias assessment for non-RCTs with the MINORS score.

	Jo et al. 2013	Taniguchi et al. 2015	Kim et al. 2020	Pulatkan et al. 2020
Clearly stated aim	2	2	2	2
Inclusion of consecutive patients	1	2	0	0
Prospective data collection	2	2	2	2
End points appropriate to study aims	2	1	2	1
Unbiased assessment of study end point	1	1	1	1
Follow-up period appropriate to study aims	2	1	2	2
Less than 5% loss to follow-up	2	2	2	2
Prospective calculation of sample size	0	0	0	0
An adequate control group	1	1	2	2
Contemporary groups	1	2	2	2
Baseline equivalence of groups	1	1	2	2
Adequate statistical analyses	2	2	2	2
Total score	17	17	19	18

### 3.4. Meta-analysis results

#### 3.4.1. Retear rates

**Figure 3:**
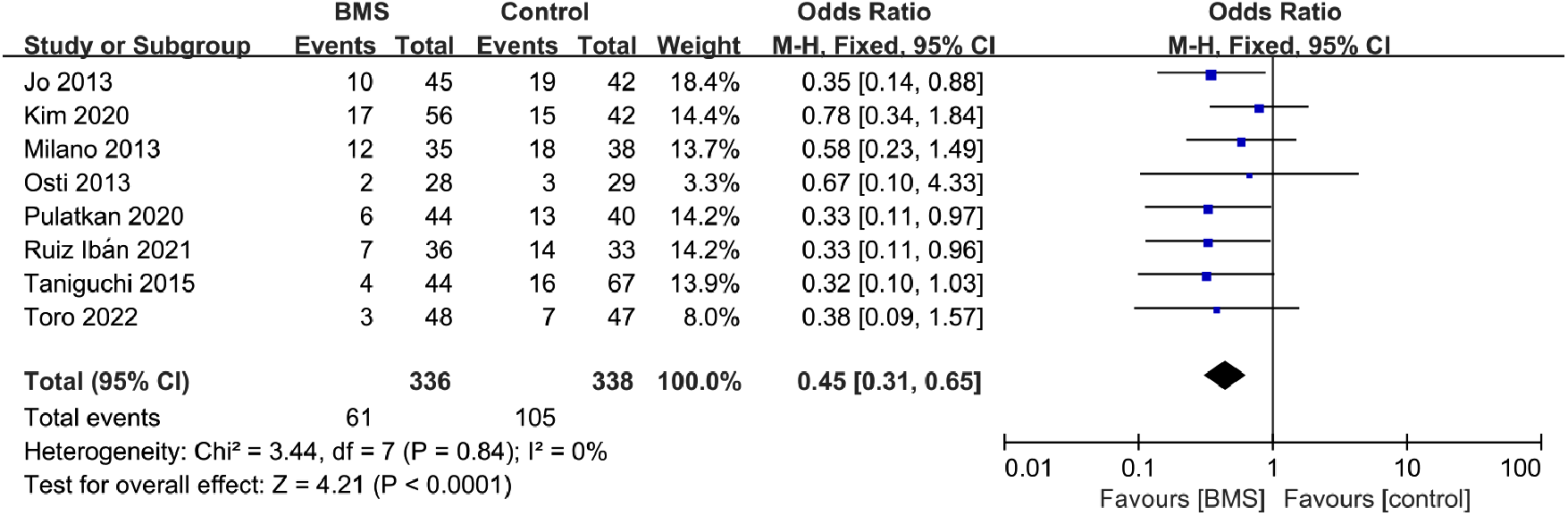
Forest plot of the incidence of retears in the BMS and control groups.

The retear was determined by postoperative computed tomography arthrography (CTA), ultrasound (US), magnetic resonance imaging (MRI) or magnetic resonance arthrography (MRA). A retear is defined when the following conditions are matched: Sugaya type IV or V appearance, modified Boileau grading system (types of retears and new tears), French Society of Arthroscopy (stage 3 and 4) or any lack of continuity in the repaired rotator cuff at follow-up. Retear rates were reported in all included studies. The retear rates for the BMS group were 18.15% (61/336), compared to 31.07% (105/338) for the control group. The pooled results from 674 patients indicated significantly lower retear rates in patients with BMS techniques than in conventional repair (OR, 0.45; 95% CI, 0.31–0.65; *P* < 0.0001; *I*^2^ = 0%) (Figure 3).

#### 3.4.2. Shoulder functional outcomes

##### 3.4.2.1. Constant score

**Figure 4:**
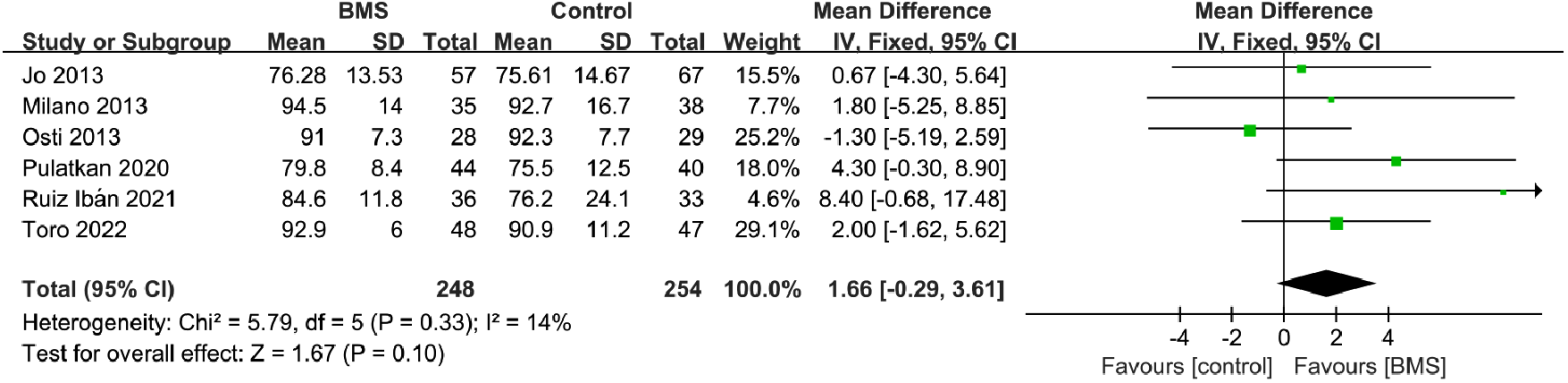
Forest plot comparing postoperative constant scores between the BMS and control groups.

A total of 6 studies with 502 patients reported the postoperative Constant score. The mean Constant score in the BMS group was 86.51, and the mean Constant score in the control group was 83.87. The pooled results showed no significant difference between the 2 groups (MD, 1.66; 95% CI, −0.29–3.61; *P *= 0.10; *I*^2^ = 14%) (Figure 4).

##### 3.4.2.2. UCLA score

**Figure 5:**
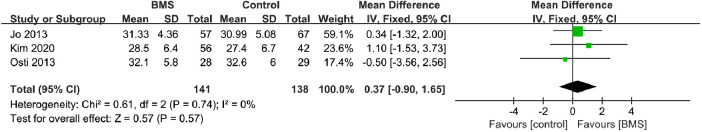
Forest plot comparing postoperative UCLA scores between the BMS and control groups.

A total of 3 studies with 279 patients reported the postoperative UCLA score. The mean UCLA score in the BMS group was 30.64, and the mean UCLA score in the control group was 30.33. The pooled results showed no significant difference between the 2 groups (MD, 0.37; 95% CI, −0.90–1.65; *P* = 0.57; *I*^2^ = 0%) (Figure 5).

##### 3.4.2.3. ASES score

**Figure 6:**
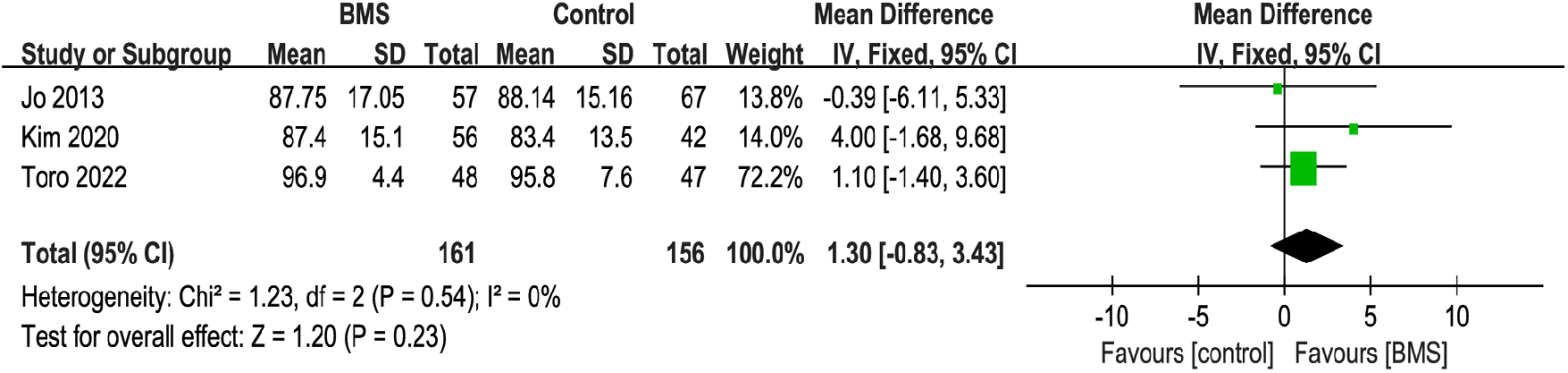
Forest plot comparing postoperative ASES scores between the BMS and control groups.

A total of 3 studies that included 317 patients reported postoperative ASES score. The mean ASES score in the BMS group was 90.68, and the mean ASES score in the control group was 89.11. The pooled results showed no significant difference between the 2 groups (MD, 1.30; 95% CI, −0.83–3.43; *P* = 0.23; *I*^2^ = 0%) (Figure 6).

##### 3.4.2.4. DASH score

**Figure 7:**
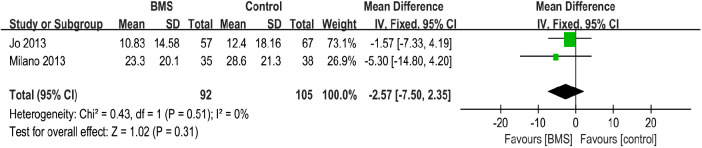
Forest plot comparing postoperative DASH scores between the BMS and control groups.

A total of 2 studies with 197 patients reported the postoperative DASH score. The mean DASH score in the BMS group was 17.07, and the mean DASH score in the control group was 20.5. The pooled results showed no significant difference between the 2 groups (MD, −2.57; 95% CI, −7.50–2.35; *P* = 0.31; *I*^2^ = 0%) (Figure 7).

#### 3.4.3. VAS score

**Figure 8:**
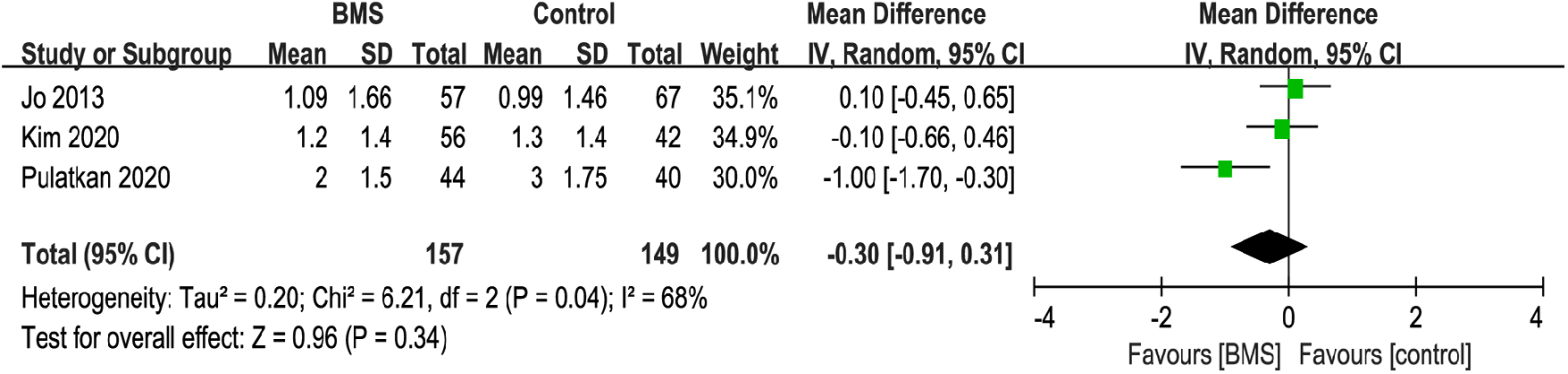
Forest plot comparing postoperative VAS score between the BMS and control groups.

A total of 3 studies with 306 patients reported the postoperative VAS score. The mean VAS score in the BMS group was 1.43, and the mean VAS score in the control group was 1.76. The pooled results showed no significant difference between the 2 groups (MD, −0.30; 95% CI, −0.91–0.31; *P* = 0.34; *I^2^* = 68%) (Figure 8). The heterogeneity among the studies was high, and sensitivity analyses were performed by sequentially removing the included studies. Heterogeneity was dramatically reduced after removing the studies by Pulatkan et al. (31) (MD, 0; 95% CI, −0.39–0.40; *P *= 1.00; *I^2^* = 0%). Based on the investigation of study characteristics, it was speculated that the main source of heterogeneity might be the difference in the depth and diameter of the holes. The diameter and depth of the holes drilled by Pulatkan et al. were significantly lower than in the other two studies, which may account for the significantly lower VAS score of the patients after surgery than the other groups.

#### 3.4.4. ROM

**Figure 9:**
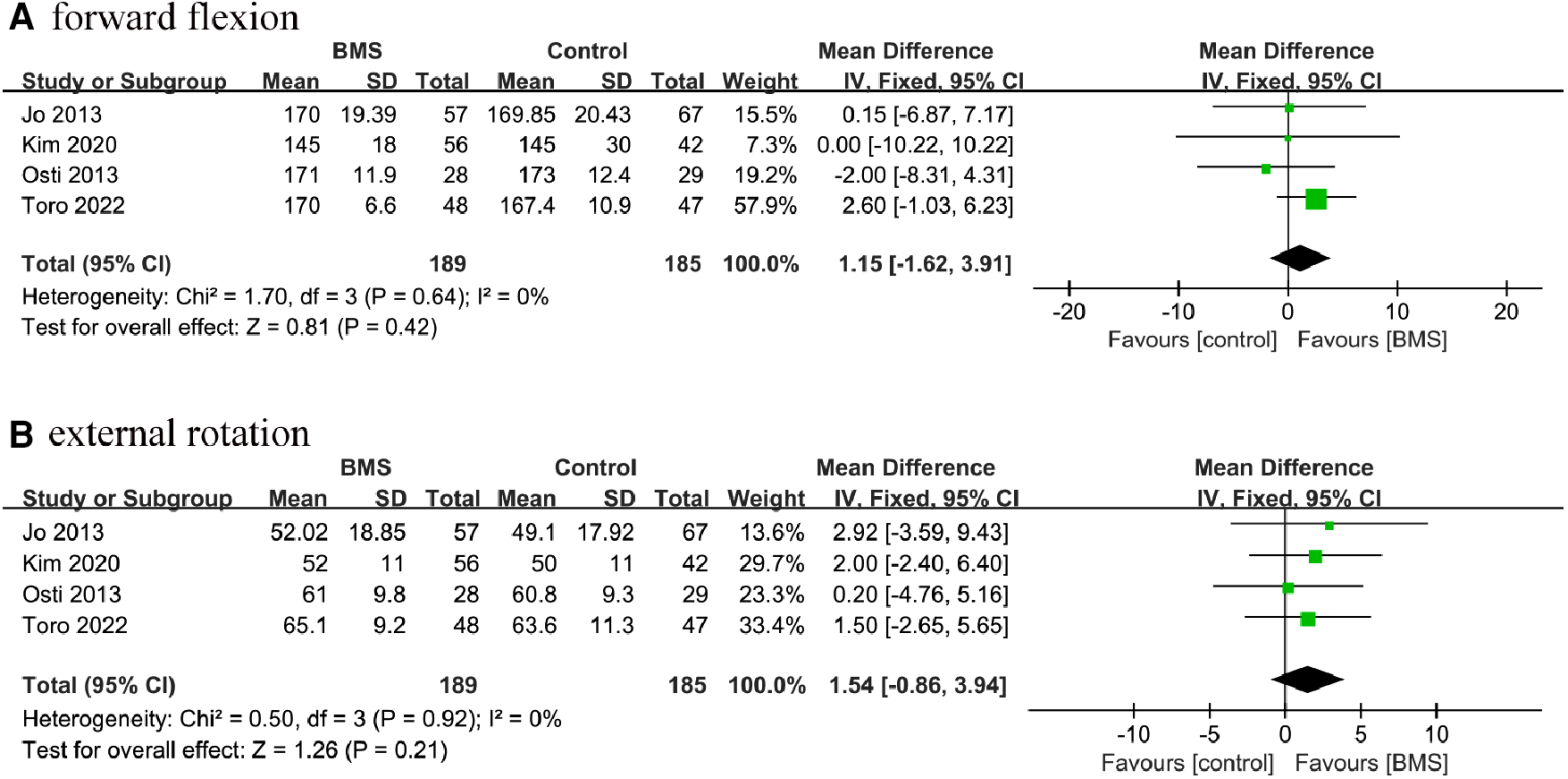
Forest plot comparing postoperative ROM between the BMS and control groups. (**A**) forward flexion; (**B**) external rotation.

A total of 4 studies with 374 patients reported postoperative ROM. Based on the available data, we conducted statistical analyses of the 2 directions of the ROM: forward flexion and external rotation. Our results showed that there were no significant differences in forward flexion (MD, 1.51; 95% CI, −1.62–3.91; *P* = 0.42; *I*^2^= 0%) or external rotation (MD, 1.54; 95% CI, −0.86–3.94; *P* = 0.21; *I^2^* = 0%) between the 2 groups (Figure 9).

### 3.5. Additional assessments

According to sensitivity analysis, all results remained robust after sequentially excluding individual study except for the Constant score. After excluding the study by Osti et al. (34) heterogeneity decreased markedly and significant difference in Constant score between the 2 groups was observed (MD, 2.65; 95% CI, 0.40–4.91; *P* = 0.02; *I*^2^ = 0%). But given that the minimal clinically important difference (MCID) of the Constant score is set to at least 10 points (35, 36), the difference between the 2 groups in this score was not clinically significant.

Although a meta-analysis of well-designed non-RCTs of surgical procedures is probably as accurate as that of RCTs (27), mixing RCTs and observational studies may skew the results. Therefore, we performed subgroup analyses for available outcomes based on study design (RCT or non-RCT), including retear rates, Constant score, UCLA score, ASES score, VAS score and ROM. The statistical results were stable and supported our conclusion favorably (Table 4). Besides, we performed subgroups analyses based on follow-up time (≤24 months and >24 months), depth and diameter of holes, and repair technique. The details of the results are summarized in Table 5.

**Table 4:** Subgroup analyses according to study design.

Outcomes	No. of studies	Study design		OR/WMD, (95%CI), *I*^2^		*P-*value	
		RCT	Non-RCT	RCT	Non-RCT	RCT	Non-RCT
Retear rate	8	4	4	0.46 [0.25,0.83], 0%	0.44 [0.27,0.71], 0%	0.01	0.0009
Constant score	6	4	2	1.17 [−1.22, 3.57], 29%	2.62 [−0.75,6.00], 9%	0.34	0.13
UCLA score	3	1	2	v0.50 [−3.56,2.56], NA	0.56 [−0.85,1.96], 0%	0.75	0.44
ASES score	3	1	2	1.10 [−1.40, 3.60], NA	1.82 [2.21,5.85], 12%	0.39	0.38
VAS score	3	0	3	NA	−0.30 [0.91,0.31],68%	NA	0.34
ROM							
Forward flexion	4	2	2	1.45 [−1.69, 4.60], 35%	0.10 [−5.68,5.89], 0%	0.37	0.97
External rotation	4	2	2	0.97 [−2.22, 4.15], 0%	2.29 [−1.36,5.93], 0%	0.55	0.22

OR, odds ratio; WMD, weighted mean difference; CI, confidence interval; RCT, randomized controlled trial; NA, not applicable; UCLA, University of California at Los Angeles; ASES, American Shoulder and Elbow Surgeons; VAS, Visual analog scale; ROM, range of motion.

**Table 5:** Subgroup analyses of retear rate.

	No. of studies	Retear rate		Odds ratio (95% CI)	*I^2^*	*P*-value
		BMS	Control			
**Follow-up period**						
≤24 months	4	31/184	52/189	0.46[0.27,0.79]	0%	0.004
>24 months	4	30/152	53/149	0.43[0.25,0.73]	0%	0.004
**Depth of hole**						
≤5 mm	3	34/137	48/117	0.47[0.28,0.81]	11%	0.006
>5 mm	4	23/155	41/154	0.46[0.25,0.83]	0%	0.01
**Diameter of hole**						
≤2 mm	4	42/171	60/153	0.51 [0.31,0.82]	0%	0.005
>2 mm	2	14/89	35/109	0.33 [0.16,0.69]	0%	0.003
**Repair technique**						
Single row	4	37/163	49/149	0.57[0.34,0.96]	0%	0.04
Double row	1	10/145	19/42	0.35[0.14,0.88]	NA	0.03
Surface-holding	1	4/44	16/67	0.32[0.10,1.03]	NA	0.06

**Figure 10:**
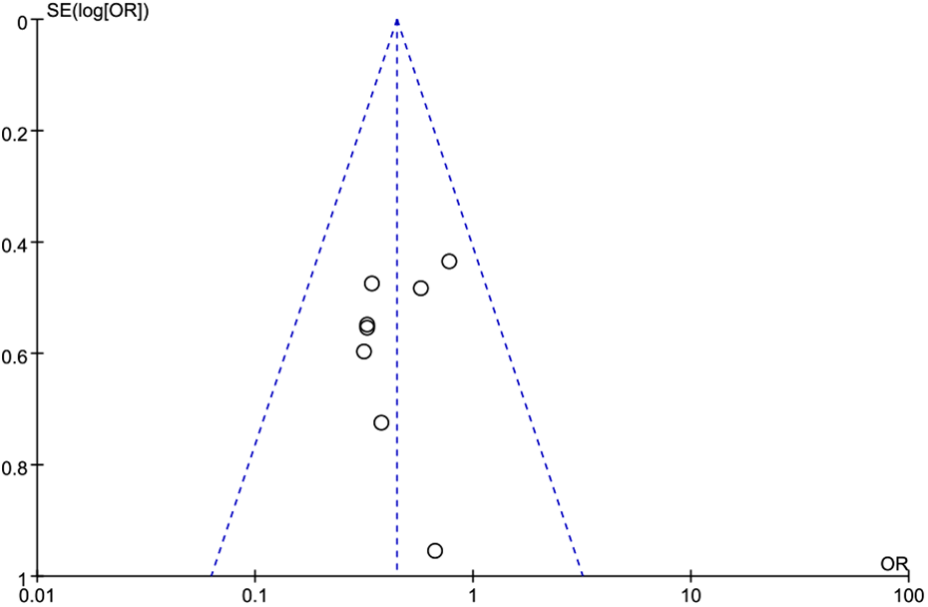
Funnel plot of data on re-tear rates.

We performed a publication bias analysis for the primary outcome-retear rate. The funnel plot for studies reporting re-tear rate data was symmetric, suggesting a low risk of publication bias (Figure 10).

## 4. Discussion

The critical finding of this study was that the combination of the BMS technique in the primary arthroscopic repair of the rotator cuff significantly reduced the retear rates. At a mean follow-up of 23.2 months, the VAS score, ROM, and functional outcomes—including the Constant score, UCLA score, ASES score, and DASH score—did not, however, show statistically significant differences between the 2 groups. These findings are similar to some studies on the effects of PRP in rotator cuff repair (10, 37, 38). The results are also in line with previous systematic reviews by Ajrawat et al. (21) and Li et al. (20). Nevertheless, we included more recent studies (19, 22, 23, 31) and excluded certain low-quality studies to improve the credibility of the results. Furthermore, we did not take the study (29) assessing concomitant BMS and patch augmentation into account.

Retear rate is one of the most important indicators to assess the success of rotator cuff tear repair and an influential point for patient satisfaction. Although previous studies have demonstrated that functional outcomes are unrelated to the structural integrity of rotator cuff repair, they have the limitation of a relatively short mean follow-up period, with the longest not exceeding 30.1 months (39–41). Recent studies with long-term follow-up have revealed the opposite results (42, 43). Jeong et al. (44) conducted a retrospective study of 201 patients with rotator cuff repair at a mean follow-up of 8.6 years. They demonstrated that functional outcomes in retear patients deteriorated over time but were unrecognized at the 2-year postoperative follow-up. At the final follow-up (>5 years postoperatively), the functional outcome of the retear group was significantly worse than the intact rotator cuff group (*P* < .001). Retears of the rotator cuff disrupt the dynamic stability of the shoulder and accelerate the progression of glenohumeral osteoarthritis, leading to worse functional outcomes. It will be a relatively long time before shoulder function deteriorates.

The mean follow-up time for our included studies was only 23.2 months, which may explain why our statistics show no significant differences in clinical outcomes between the 2 groups. Notably, most rotator cuff retears occur within 6 months of surgery, and radiographic evaluation of the repaired tendon at 6 months postoperatively is sufficient to be a reliable predictor of retear rates at long-term follow-up (45–47). Each of the four imaging modalities—MRI, MRA, CTA, and US—has been shown to be equally accurate and reliable in determining the condition of the rotator cuff (48). Therefore, it can be hypothesized that additional BMS procedures are conductive to maintaining the structural integrity of the repaired tendon and cause better clinical outcomes in long-term follow-up. But more studies with long-term follow-up are warranted to demonstrate this effectiveness.

BMS has been proven to be a well-established treatment for osteochondral lesions of the knee (49) and ankle (50). When evaluating the clinical outcomes of different knee cartilage restoration techniques, microfracture are often used as a control group to compare with other techniques, including autologous chondrocyte implantation (ACI) and osteochondral autograft transfer (OAT). Several studies (51–53) have shown that these techniques provide similar clinical benefits as microfracture. A meta-analysis by Gou et al. (51) involving 659 patients with knee cartilage lesions found no significant differences in functional outcomes of ACI compared to microfracture at 1 to 5 years of follow-up. Another meta-analysis by Mundi et al. (52) also suggested that there were no significant differences between microfracture, ACI, and OAT in improving function and pain at intermediate-term follow-up.

BMS, also described as “microfracture,” “multiple channeling,” and “Crimson Duvet,” has gained increasing attention for its utility in enhancing rotator cuff repair. The rationale for BMS is to induce multiple fractures of the greater tuberosity of the proximal humerus, which leads to the release of bone marrow mesenchymal stem cells (BMSCs), growth factors and the formation of blood clots (54, 55). Since the shoulder cartilage is not as thick as the knee joint, it is more difficult for the blood clot to stay *in situ*. Less weight bearing, however, may aid to achieve the optimal healing effect (56). Jo et al. (28) reported that the proximal humeral greater tuberosity contains typical characteristic BMSCs. In addition, Kida et al. (57) showed the efficacy of BMS here using bone marrow chimeric rats specifically expressing the green fluorescent protein in bone marrow-derived cells. They demonstrated that bone marrow-derived cells passed through holes drilled into the greater tuberosity, were recruited to the surface of the footprint and promoted rotator cuff healing. Experiments with rabbit models have shown that microfractures in rotator cuff repair promoted tendon healing and significantly increased tendon biomechanical properties with thicker collagen bundles (16). Microfracture in isolation is also an optional treatment for glenohumeral osteoarthritis or cartilage defects. Considering that the incidence of these 2 diseases in patients with rotator cuff tears ranges from 12.5% to as high as 28% (58), concurrent operations can be beneficial for a substantial portion of these patients.

However, there is a lack of a standard protocol for BMS application in the arthroscopic repair of rotator cuff patients. The diameter and depth of the drill hole may affect the clinical benefit. Sun et al. (59) investigated the impact of microfractures with various sizes on repair in rabbit rotator cuff tear models. They found that the control group without microfractures showed superior biomechanical properties compared to the large microfracture (1 mm) group, but inferior biomechanical properties compared to the small microfracture (0.5 mm) group. The large-diameter microfractures lead to subchondral collapse or failure of remodeling and worsen the healing process. This result is consistent with previous studies of cartilage defect treatment (60, 61). It is crucial to maintain a balance between promoting tendon healing and the risk of anchor loosening and damaging the vascular supply of the greater tuberosity. Among the 8 studies we included, the BMS group did not lead to inferior outcomes or complications. Theoretically, narrow and deep holes reflecting the physiological subchondral trabecular distance are sufficient to stimulate bone marrow release while preventing anchor failure (62). By microstructural analysis of the humeral tuberosity in patients with rotator cuff tears, Sakamoto et al. found that the average minimum distance between the trabecular separation was 0.7 mm (63). Therefore, based on similar studies on the knee, we speculate that small holes with a diameter of 0.7 mm are a better option for ARCR. However, there are no studies comparing the effect of different diameter holes on rotator cuff repair in humans. High-quality RCTs must be conducted to explore specifics of the BMS method. Furthermore, the BMSCs induced by microfractures are not completely retained on the surface of the tendon-bone and are partially lost to the surrounding tissue, which would compromise the effectiveness of the BMS. Yoon et al. (29) designed a novel repair technique that combined BMS and patch augmentation to enrich BMSCs and improve initial mechanical properties. Their results showed that this concomitant procedure significantly reduced retear and medial-row failure rates in the arthroscopic repair of massive rotator cuff tears.

Overall, BMS is a straightforward and safe technique that can promote rotator cuff healing and slow the progression of osteoarthritis. It does not require additional costs or particular instruments. Even for massive tears, it can be completed in approximately 10 min (18). Currently, BMS is a viable and effective method for promoting tendon healing as compared to alternative biological repair techniques, which are expensive or have undetermined side effects.

## 5. Limitations

There were some noted limitations of this review. First, half of the 8 studies included were non-RCTs, possibly compromising the credibility due to selection bias. Although the subgroup analyses based on the study design demonstrated the robustness of the results, the findings should be interpreted with caution. Second, some baseline characteristics including fixation method, tear size, rehabilitation protocol, parameters of the BMS technique, varied across studies, and these factors could affect clinical outcomes. Thirdly, analyses of long-term clinical outcomes were not possible due to the short average follow-up period of the included trials. Fourth, some risk factors affecting postoperative outcomes, including smoking, body mass index and diabetes, were not documented in the included studies and may contribute to confounding bias.

## 6. Conclusion

Compared to ARCR alone, the combination of intraoperative BMS technique can significantly reduce the retear rates, but showed similar short-term results in functional outcomes, ROM and pain. Better clinical outcomes are anticipated in the BMS group by improving structural integrity during long-term follow-up. Future studies are also encouraged to investigate standard parameters of BMS, such as depth, diameter and drilling method, which may affect repair outcomes. The current results show that the smaller diameter of the hole can achieve the desired effect without negatively affecting the function. BMS may be a viable option in ARCR based on its straightforward and cost-effective advantages.

## Data Availability

The original contributions presented in the study are included in the article/Supplementary Material, further inquiries can be directed to the corresponding author/s.
